# Prevalence and associated risk factors for *Salmonella enterica* contamination of cow milk and cottage cheese in Ethiopia

**DOI:** 10.1186/s40550-023-00101-3

**Published:** 2023-02-17

**Authors:** Abdi Bedassa, Henok Nahusenay, Zerihun Asefa, Tesfaye Sisay, Gebrerufael Girmay, Jasna Kovac, Jessie L. Vipham, Ashagrie Zewdu

**Affiliations:** 1 Ethiopian Institute of Agricultural Research, National Agricultural Biotechnology Research Center, PO Box 249, Holeta, Ethiopia; 2 Ethiopian Institute of Agricultural Research, Megenagna Near Egziarab Church, P.O.Box: 2003, Addis Ababa, Ethiopia; 3 Ethiopian Institute of Agricultural Research, Holeta Agricultural Research Centre, PO Box 031, Holeta, Ethiopia; 4 Institute of Biotechnology, College of Natural and Computational Sciences, New Graduate Building, Addis Ababa University, P.O. Box 1176, Addis Ababa, Ethiopia; 5 Department of Food Science, The Pennsylvania State University, 437 Erickson Food Science Building, University Park, State College, PA 16802 USA; 6 Department of Animal Sciences and Industry, Kansas State University, 247 Weber Hall, Manhattan, KS 66506 USA; 7 Center for Food Science and Nutrition, Addis Ababa University, New Graduate Building, College of Natural Sciences, P.O. Box 1176, Addis Ababa, Ethiopia

**Keywords:** Cottage cheese, Raw milk, Pasteurized milk, Risk factors, *Salmonella enterica*

## Abstract

Animal sourced foods, such as dairy products, are common sources of *Salmonella enterica*, a foodborne pathogen of increasing global concern, particularly in developing countries. In Ethiopia, most data on the prevalence of *Salmonella* in dairy products is highly varied and limited to a specific region or district. Furthermore, there is no data available on the risk factors for *Salmonella* contamination of cow milk and cottage cheese in Ethiopia. This study was therefore conducted to determine the presence of *Salmonella* throughout the Ethiopian dairy value chain and to identify risk factors for contamination with *Salmonella*. The study was carried out in three regions of Ethiopia, including Oromia, Southern Nations, Nationalities and Peoples, and Amhara during a dry season. A total 912 samples were collected from milk producers, collectors, processors, and retailers. Samples were tested for *Salmonella* using the ISO 6579-1: 2008 method, followed by PCR confirmation. Concurrent with sample collection, a survey was administered to study participants to identify risk factors associated with *Salmonella* contamination. *Salmonella* contamination was highest in raw milk samples at the production (19.7%) and at milk collection (21.3%) levels. No significant difference in the prevalence of *Salmonella* contamination among regions was detected (*p* > 0.05). Regional differences were apparent for cottage cheese, with the highest prevalence being in Oromia (6.3%). Identified risk factors included the temperature of water used for cow udder washing, the practice of mixing milk lots, the type of milk container, use of refrigeration, and milk filtration. These identified factors can be leveraged to develop targeted intervention strategies aimed at reducing the prevalence of *Salmonella* in milk and cottage cheese in Ethiopia.

## Introduction

Foodborne disease is a global issue, with approximately 600 million cases of illness and 420,000 deaths associated with foodborne pathogens on an annual basis (Havelaar et al., 2015). In low- and middle-income countries, the problem is often more severe and less documented compared to high-income countries (Wabeto et al.*,*^2017^). However, anecdotal data indicate that this is due to poor food handling and sanitation practices, inadequate food safety laws, weak regulatory systems, lack of financial resources, and poor awareness of proper food handling practices (Asfaw Geresu et al., 2021). Among the common bacterial foodborne pathogens, non-typhoidal *Salmonella enterica* (*Salmonella*) is considered a pathogen of high public health concern (Chen et al., 2013; Henry et al., 2017).

Although many countries have milk safety regulations and surveillance systems for monitoring of foodborne pathogens to ensure food safety, such surveillance of milk and milk products is not conducted on a routine basis in Ethiopia. As a result, there is limited information available on the microbial safety of milk (Bereda et al., 2014; Birke and Zewide, 2019). This is particularly true in the central highlands of Ethiopia, where milk production plays a significant role in the diets and economies of communities (Tegegne et al., 2013; Gizaw et al., 2016). Previous studies on the prevalence of *Salmonella* in dairy products in Ethiopia have reported a range of prevalence, between 6.5% to 20% in milk, and between 0% to 3.1% in cottage cheese (Tesfaw et al., 2013; Tadesse and Dabassa, 2012; Ejo et al., 2016; Beyene et al., 2016;). These studies have been conducted in different regions of Ethiopia and samples have been collected at different points in the dairy value chain. However, to date, no study has systematically investigated differences in the prevalence of *Salmonella* among major milk producing regions in Ethiopia, nor among different points in the value chain, from production, collection, processing, to retail.

In addition to prevalence, understanding the factors that increase the odds of *Salmonella* contamination of milk and cottage cheese is critically important for the development of effective mitigation strategies in Ethiopia. Past studies that have assessed the hygiene of milk containers, the environments in which milking takes place, the bedding in cow barns, the quality of the feed, and the hygiene of people engaged in milking have already identified inadequate practices as likely contributors to poor milk quality/safety (Amanuel and Ulfina, 2018; Adugna and Eshetu, 2021). However, to the best of our knowledge, no study has identified risk factors associated with *Salmonella* contamination of milk and milk products in Ethiopia (Keba et al., 2020).

This study aimed to fill the above-outlined gaps in the knowledge by determining the prevalence of *Salmonella* in milk and cottage cheese along milk and cottage cheese value chains in three major milk-producing regions of Ethiopia, including Oromia, Southern Nations, Nationalities and Peoples (SNNP), and Amhara. Furthermore, it aimed to identify risk factors for contamination with *Salmonella* that could inform the development of interventions.

## Methods

### Study areas and study design

**Fig. 1:**
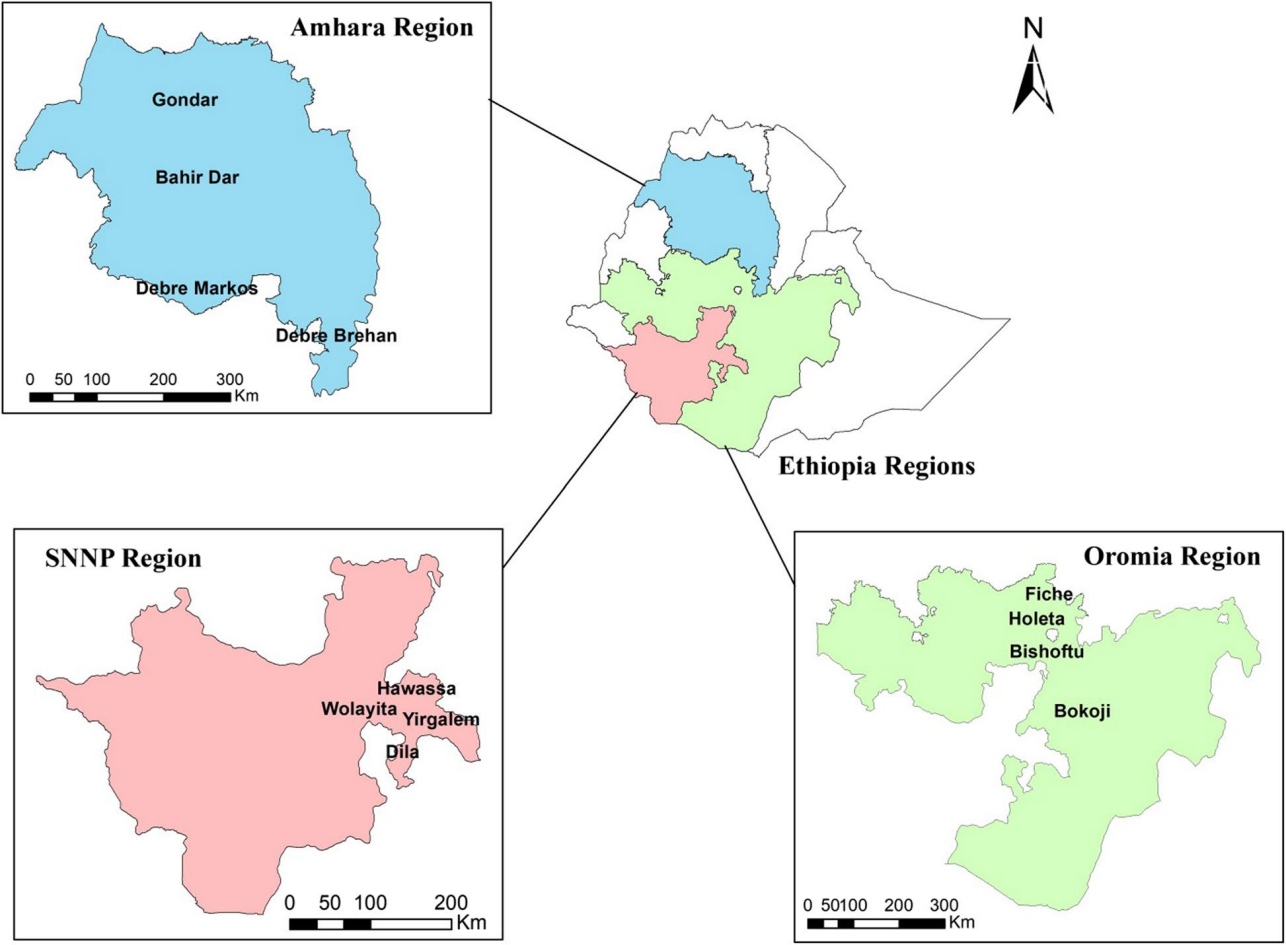
Map of the study areas. Study sites within each region are listed on maps of indvidual regions

This study was conducted in three regions of Ethiopia (Fig. 1) based on the dairy production potential of the country (CSA, 2019). According to Central Statistical Agency (CSA) data (2010), not all regions have the same capacity to produce cow milk. The estimated milk production potential in Oromia is 52%, in SNNP 23%, in Amhara 20%, and in Tigray 5% of Ethiopia’s total milk production volume. This information was used to estimate the minimum sample size for each region, which was determined to be 480 in Oromia, 240 in SNNP, and 194 in Amhara. Samples were ultimately not collected in Tigray due to safety concerns related to a political unrest.

A cross-sectional study was conducted during a dry season between December 2020 and May 2021 to determine the prevalence of *Salmonella* along the milk and cottage cheese value chains in each region. After the sites selection, product-based strata were created based on the milk production potential and availability of participants for each level in a value chain. A list of milk producers, collectors, processors, and retailors was obtained from each study area representatives (i.e., extension agents). Participants were randomly selected for inclusion in the study from the list of potential participants.

### Sample collection

Approximately 250 mL of raw and pasteurized milk were aseptically collected and placed in a sterile polyethylene bottle and 500 g of cottage cheese samples were collected in a sterile polyethylene zip-lock bag and stored in a portable cooler at 4 °C. Samples were transported in a cooler to the National Agricultural Biotechnology Research Center, Microbial Biotechnology Research Laboratory, and stored at 4 °C until analyzed. Samples were analyzed within 12 hours after delivery to the laboratory.

### *Salmonella* enrichment, isolation, and confirmation

*Salmonella* enrichment and isolation were completed according to ISO 6579-1: (ISO 2017; Mooijman, 2018) and putative *Salmonella* isolates were PCR-confirmed (Andrews et al., 2018). Briefly, 25 mL of milk or 25 g of cottage cheese were transferred to 225 ml of sterile buffered peptone water (Oxoid, CM 0509), homogenized in a stomacher bag, and incubated at 35 °C for 18 hours. After incubation, 0.1 mL and 1 mL of a primary enrichment was aseptically inoculated into 10 mL of sterile Rappaport Vassiliadis (RV) broth (HiMedia) and Muller Kaufmann Tetrathionate (MKTTn) broth (HiMedia), respectively. An inoculated RV broth was incubated at 41 °C for 24 h and inoculated MKTTn broth was incubated at 37 °C for 24 h. A 10 μl loopful of cultured RV and MKTTn broths was streaked onto both Xylose Lysine Deoxycholate (XLD) agar (HiMedia) and Hektoen Enteric (HE) agar (HiMedia). Inoculated plates were incubated aerobically at 37 °C for 24 h. Typical pink or red colonies with or without black centers on XLD and blue-green to blue colonies with or without black centers on HE agar were sub-cultured on BHI agar (BBL) and incubated at 35° ± 2.0 °C for 24 ± 2.0 h for molecular confirmation.

### Molecular confirmation

*Salmonella* isolates were confirmed utilizing PCR targeting the *invA* gene (Galán and Curtiss 3rd., 1991). The extraction of DNA was performed by thermal cell lysis at 95–100 °C for 10 min (El-baz et al., 2017; Reischl and Wittwer, 2001). Extracted DNA was used as a template for the amplification of highly conserved region of *invA* gene using primers *Salm3* (5′-GCTGCGCGCGAACGGCGAAG-3′) and *Salm4* (5′-TCCCGGCAGAGTTCCCATT*-*3′) that amplify a 389 bp fragment of the conserved *invA* gene sequence of *Salmonella.*

PCR included initial denaturation at 95 °C for 5 min followed by 35 cycles of amplification (denaturation at 95 °C for 90 s, annealing at 60 °C for 60 s, and extension at 72 °C for 90 s), ending with a final extension at 72 °C for 7 min, using a BioRad T100 thermocycler. PCR amplicons were confirmed with gel electrophoresis using a 1.5% w/v agarose gel. Gels were examined using an UV transillumination system (UVP BioDoc-It Imaging Systems, Analytik Jena) (Shanmugasamy et al.*,*^2011^; El-baz et al.*,*^2017^).

### Survey data collection

Data on milk and cottage cheese production, storage, transportation, and packaging operations was collected by a pre-tested semi-structured questionnaire approved by the Institutional Review Board of the Addis-Ababa University (CNS-IRB 42/2019). The survey was administered using the Kobo toolbox. Aside from providing a questionnaire, a direct examination of general cleanliness, sanitary practices, and pasteurized milk and cottage cheese packaging materials were conducted and recorded. The survey was administered at the time of milk or cottage cheese sample collection for laboratory analyses for *Salmonella.*

### Statistical analysis

Binomial response variable (i.e., presence or absence of *Salmonella*) was considered as an outcome variable and the region, product type, points in the value chain were considered as explanatory (independent) variables. As the outcome was dichotomous, a logistic regression model was used to analyze the association of the outcome variable with the explanatory variables. Odds ratio (OR) was used to measure the degree of association among and within the explanatory variables. In all analyses, alpha was set to 0.05. For all the analysis, Stata version 16 software was used using the following formula:

$${P}^{\hat{\mkern6mu} }=\frac{Exp\Big( bo+b1X1+b2X2+\dots + bpXp}{1+\left( Exp\right( bo+b1X1+b2X2+\dots + bpXp}$$

Where P^ is the expected probability that the outcome (*Salmonella* contamination) is positive; X1 through Xp are distinct independent variables, and b0 through bp are the regression coefficients.

## Results

### Prevalence of *Salmonella* in raw milk, pasteurized milk, and cottage cheese

Table 1 presents the prevalence of *Salmonella* between pasteurized and raw milk in each study regions. *Salmonella* prevalence differed significantly between raw and pasteurized milk only in the Oromia region (*p* = 0.015). Furthermore, the prevalence of *Salmonella* in raw milk was highest in Oromia (21.4%), followed by SNNPs (19.8%), and Amhara (14.1%); however, these differences were not statistically significant (Table 2). Similarly, the prevalence of *Salmonella* in samples from pasteurized milk was highest in Oromia (12.5%), followed by SNNP (11.5%), and Amhara (9%), but the differences were not statistically significant (Table 3).

**Table 1:** Prevalence of *Salmonella* in raw and pasteurized milk samples collected in each study region in Ethiopia

Region	Milk type	Sample size	*Salmonella* positive (n)	*Salmonella* positive (%)	OR^a^	χ^2 b^	*P*-value
Oromia	Raw milk	192	42	21.8	1.6	5.9	0.015
	Pasteurized milk	192	24	12.5	1		
	Total	384	66	17.2			
SNNPs	Raw milk	96	19	19.79	1.5	2.5	0.112
	Pasteurized milk	96	11	11.45	1		
	Total	192	30	15.6			
Amhara	Raw milk	80	11	14.1	1.4	4.0	0.267
	Pasteurized milk	76	7	9.2	1		
	Total	156	18	11.5			

^a^*OR* Odds ratio

^b^χ^2^, chi-square

**Table 2:** Overall prevalence of *Salmonella* in raw milk by region and within value chain

Variable	Observation	Sample size	*Salmonella* positive (n)	*Salmonella* positive (%)	χ^2 a^	*P* value
Region	**Amhara**	78	11	14.1	1.9	0.391
	**Oromia**	192	41	21.4		
	**SNNPs**	96	19	19.8		
	**Total**	366	71	19.4		
Value chain	**Producer**	183	33	18.0	0.4	0.509
	**Collector**	183	38	20.8		
	**Total**	366	71	19.4		

^a^χ^2^, chi-square

**Table 3:** Overall prevalence of *Salmonella* in pasteurized milk by region and within value chain

Variable	Observation	Sample size	*Salmonella* positive (n)	*Salmonella* positive (%)	χ^2 a^	*P* value
Region	**Amhara**	78	7	9.0	0.7	0.712
	**Oromia**	192	24	12.5		
	**SNNPs**	96	11	11.5		
	**Total**	366	42	11.5		
Value chain	**Processor**	183	25	13.7	1	0.190
	**Retailer**	183	17	9.3		
	**Total**	366	42	11.5		

^a^χ^2^, chi-square

The prevalence of *Salmonella* in cottage cheese was 6.3% in samples collected from producers, as well as from retailers in Oromia. No *Salmonella* was detected in cottage cheese samples collected from retailers in Amhara nor in SNNP (Table 4). The differences in prevalence between producers and retailers within each region were not statistically significant, as shown in Table 4. The overall total prevalence among the raw and pasteurized milk was about 15.6% (114/732) throughout the study areas of the country. It was higher in the raw milk type with 19.7% (72/366) and lower in pasteurized milk with 11.5% (42/366), as shown in Table 5.

**Table 4:** Prevalence of *Salmonella* in cottage cheese samples collected from producers and retailers in three study regions in Ethiopia

Region	Value chain	Sample size	*Salmonella* positive (n)	*Salmonella* positive (%)	χ^2 a^	*P* value
Oromia	Producer	48	3	6.25	0	1.000
	Retailer	48	3	6.25		
	Total	96	6	6.25		
SNNPs	Producer	24	1	4.2	1.4	0.235
	Retailer	24	0	0.0		
	Total	48	1	2.1		
Amhara	Producer	16	1	6.2	1	1.000
	Retailer	16	0	0.0		
	Total	32	1	3.1		

^a^χ^2^, chi-square

When comparing *Salmonella* prevalence along the value chain, milk samples taken from collectors had the highest prevalence of *Salmonella* (21.3%; *p* = 0.01), whereas samples collected from retailers had the lowest prevalence (9.3%). Prevalence of *Salmonella* in samples collected from producers was lower (18.1%) compared to those collected from collectors, but higher compared to those collected from processors (13.7%; *p* = 0.01) (Table 5). The overall prevalence of *Salmonella* was highest in Oromia (17.2%), followed by SNNP (15.6%), and Amhara (11.5%); however, the differences in the prevalence among regions were not statistically significant (*p* = 0.26) (Table 5).

**Table 5:** Overall total prevalence of *Salmonella* in raw and pasteurized milk, along a value chain, and in different regions of Ethiopia

Variable	Observation	Sample size	*Salmonella-*positive (n)	*Salmonella-*positive (%)	OR^a^	χ^2 b^	*P*-value
Milk Type	Raw milk	366	72	19.7	1.6	9.4	0.002
	Pasteurized milk	366	42	11.5	1		
	Total	732	114	15.6			
Value chain	Producer	183	33	18.1	1.8	11.4	0.01
	Collector	183	39	21.3	2		
	Processor	183	25	13.7	1.4		
	Retailer	183	17	9.3	1		
	Total	732	114	15.6			
Region	Oromia	384	66	17.2	1.4	2.7	0.260
	SNNPs	192	30	15.6	1.3		
	Amhara	156	18	11.5	1		
	Total	732	114	15.6			

^a^OR, odds ratio

^b^χ^2^, chi-square

### Risk factors associated with *Salmonella* contamination of milk and cottage cheese

It is important to note that this assessment has evaluated more variables than presented in Table 6, however, many of these tested variables were statistically insignificantly. Therefore, only variables with statistical significance are summarized in Table 6 to ease the readability and data interpretation.

#### Risk factors for milk contamination at the production level

Among 184 surveyed milk producers, 32.6% lived in urban and 67.4% in peri-urban areas. The gender ratio among surveyed participants was nearly equal (52.2% men and 47.8% women). Among surveyed farmers, the majority had only informal education (49.6%), followed by elementary school (52.8%), high school (40.7%), preparatory (13.7%), and a college degree (30.3%). Surveyed participants were asked about hygiene practices carried out at the farm. Survey results showed that washing udders of cows before milking was performed by 94% of farmers. Sixty-seven percent of the surveyed farmers also dried washed udders utilizing pieces of clothes. The above outlined variables were not significantly associated with the prevalence of *Salmonella* (*p* > 0.05).

Based on the survey and microbial data, a lower prevalence of *Salmonella* was observed in raw milk collected from untrained study participants of milk hygienic handling-related training, while milk collected from trained study participants had a higher prevalence of *Salmonella* (25.2%; *p* = 0.002). Milk collected from individuals that used cold water for cleaning cow udder was more frequently contaminated with *Salmonella* (25.8%) compared to milk collected from individuals utilizing hot water (13.1%; *p* = 0.03). All milk samples collected from producers that mixed milk collected in the morning and evening were contaminated with *Salmonella*, whereas *Salmonella* was detected in just 16.7% of samples from participants that did not mix morning and evening milk batches (*p* = 0.029). Finally, *Salmonella* prevalence in milk collected from participants that stored milk in aluminum cans was higher (46.1%) compared to those that stored milk in plastic cans (15.2%) and mazzi cans (14.3%; *p* = 0.018).

Our data indicate that training related to milk handling, water temperature used for udder washing, mixing of evening and morning batches of milk, and types of milk handling containers all had a significant effect on the prevalence of *Salmonella* in milk (Table 6)*.* Based on study participants’ responses, most producers had access to training related to milk production and hygienic milk handling (e.g., housing, feeding, milking, and health issues). Surprisingly, data analysis indicated a higher prevalence of *Salmonella* in products collected from respondents that attended a training compared to respondents that did not (*p* = 0.002). It is, however, important to note that this study did not evaluate the curriculum nor the effectiveness of these trainings, making it difficult to make inferences about this counter-intuitive finding. Further research on this topic could help to reveal the factors that may be contributing to this outcome. Finally, the practice of mixing milk from evening and morning milking showed a greater association with *Salmonella* contamination than in those who do not (*p* = 0.029; Table 6).

**Table 6:** Risk factors associated with *Salmonella* contamination at milk production and collection level

Value chain	Variable	Response	N^**a**^	n (%)^**b**^	95% CI^**c**^ of AP^**d**^	OR^**e**^	χ^**2 f**^	***p***-value
**Milk producers**	Have you ever attended food safety training?	Yes	103	26 (25.2)	17.2–34.7	2.7	9.8	0.002
		No	81	6 (7.4)	2.7–15.4	1		
		Total	184	32 (17.4)	12.2–23.6			
	What water temperature do you use for cow udder washing?	Cold	62	16 (25.8)	15.5–38.5	1.7	4.5	0.03
		Warm	115	15 (13.1)	7.5–20.6	1		
		Total	177	31 (17.5)	12.2–23.9			
	Do you mix milk from a diseased animal with that from healthy ones?	Yes	1	1 (100.0)	2.5	7.2	4.7	0.029
		No	102	17 (16.7)	10.0–25.3	1		
		Total	103	18 (17.5)	10.7–26.2			
	What type of milk handling container do you use?	Aluminum can	13	6 (46.1)	19.2–74.8	2	8.1	0.018
		Mazzi can	7	1 (14.3)	0.3–57.8	1		
		Plastic container	164	25 (15.2)	10.1–21.7	1.1		
		Total	184	32 (17.4)	12.2–23.6			
**Milk collectors**	How do you refrigerate milk?	Bulk tankers	4	1 (5.0)	0.1–24.8	1	6.5	0.039
		Deep freezers (−20 °C)	4	7 (16.7)	6.9–31.4	3		
		Refrigerators (+ 4 °C)	29	17 (30.4)	18.8–44.1	4.5		
		Total	37	25 (21.2)	14.6–26.6			
	What type of milk handling containers do you use?	Aluminum cans	15	21 (20.8)	13.3–30.0	1.1	8.6	0.013
		Plastic containers	31	9 (13.2)	6.2–23.6	1		
		Both	12	7 (46.7)	21.3–73.4	1.6		
		Total	58	37 (20.1)	14.6			
	Do you filter milk?	Yes	49	29 (25.0)	17.4–33.9	1.8	4.6	0.031
		No	9	8 (11.8)	5.2–21.8	1		
		Total	58	37 (20.1)	14.6–26.6			

^a^N, sample size

^b^n (%), number of positive isolates (percentage of postive isolates)

^c^CI, confidence interval

^d^AP, apparent prevalence

^e^OR, odds ratio

^f^ χ^2^, chi-square

#### Risk factors for milk contamination at the collection level

A total of 58 milk collectors participated in the survey, with 79.3% of them living in urban areas. Among surveyed individuals, there were 51.7% women and 48.3% men. Approximately a quarter of participants (24.2%) had primary school education, 36.2% completed high school and had a diploma/college degree, and 8.6% completed preparatory school. One third of the respondents had 1–2 or 2–5 years of experience collecting milk. According to the survey, 65.9% of study participants collected milk by foot, while 20.3% used a three-wheel drive and 13.8% used a four-wheel drive. In terms of cooling systems used during transportation of milk to collection centers, 63.8% of surveyed individuals indicated that they had a cooling system (e.g., refrigerator). At the collection center, almost all (91.4%) of surveyed participants indicated that they store milk containers on a concrete floor, and 96.5% used tap water to clean the containers. The above outlined factors were not statistically significantly associated with *Salmonella* prevalence (*p* > 0.05).

Refrigeration system, milk containers, and milk filtration used by collectors were significantly and positively associated with *Salmonella* contamination (Table 6). In contrast, *Salmonella* prevalence was significantly lower in milk samples collected from collectors that use refrigerated bulk tanks (5%) but was higher for users of + 4 °C refrigerators (30.4%) and for deep freezer (− 20 °C) users (16.7%) (*p* = 0.039). The prevalence of *Salmonella* was significantly higher in milk samples collected from collectors that used both plastic containers and aluminum cans compared to those that used mazzi can (*p* = 0.013). Lastly, milk filtration was associated with increased prevalence of *Salmonella*. Specifically, milk collected from collectors that filtered milk was two times more likely to be contaminated with *Salmonella* compared to milk collected from collectors that did not filter milk (*p* = 0.031).

#### Risk factors for milk contamination at processing level

A total of 12 milk processors were surveyed in this study. Seventy-five percent of the participants lived in urban areas and 92% were men. Nearly half of them (42%) had more than 10 years of experience with milk processing, 17% had less than a year of experience, 16% had 5–10 years of experience and 25% had 2–5 years of experience. Almost all participating processors (92%) have had had basic food safety training. Most participants (67%) cleaned milk containers twice a day, while the rest did so once a day. The majority of respondents (67%) used tap water for cleaning milking equipment, while the rest used groundwater. Twenty-five percent of the respondents used a piece of cloth to sieve the milk, while 67% of them used microfiltration and 8% used a metal sieve. Almost all (92%) of the respondents reported having cold storage for pasteurized milk. None of these variables had a significant effect on the prevalence of *Salmonella*.

Factors associated with an increased *Salmonella* prevalence at the milk processing level included sanitation and inspection procedures. For example, it revealed the prevalence had doubled in the evaluators of tests that include: alcohol, organoleptic, Lacto scan, and microbiology compared with alcohol, organoleptic and microbiological test analyzers (excluding Lacto scan in the latter). It was almost the same as only alcohol and organoleptic test assessors (*p* = 0.006). It was important to point out that not all processors used the same factors. Consequently, these processors were classified based on their various testing methodologies, and the results were analyzed accordingly.

#### Risk factors for milk contamination at the retail level

A total of 181 milk retailers from Oromia, Amhara, and SNNP were interviewed. Fifty-three percent of the respondents were male. The assessment of the educational background revealed that 3.3%, 24.3%, 30.4%, 8.3%, and 33.7% had informal, primary school, high school, preparatory, and college diploma/degree, respectively. The majority of the respondents (79.6%) had less than 5 years of experience in retail. Almost none of the respondents had access to hygienic/safe milk handling training.

Over half of surveyed participants (51.4%) acquired milk from whole sellers, 45.8% from factories, and 2.8% at the factories gate. Most surveyed retailers (78.3%) used trucks with cooling systems to deliver milk to the retailing station, whereas around 21.7% used four-wheel vehicles without a cooling system. A temperature-controlled cooling system was used by 49.7% of retailers. The majority of retailers (70.7%) had a separate refrigerator for milk storage until sale. None of the above-listed factors were statistically significantly associated with *Salmonella* contamination.

#### Risk factors for contamination of cottage cheese at the production level

A total of 89 cottage cheese producers were surveyed and 56% of them were from the peri-urban area. Ninety-two percent were women. Around one-third of the respondents were illiterate and 30% had primary school education, 27% had high school education, and 5% had preparatory school education and had diplomas/degrees. The respondents’ experience in years was less than a year, 1–2 years, 2–5 years, 5–10 years, and more than 10 years for 7%, 20%, 32%, 15%, and 27% of respondents, respectively.

Almost all of the surveyed cottage cheese producers (92%) received training on proper handling and storage of cottage cheese. The cottage cheese was placed in whey by 60% of the respondents, while 34% used a refrigerator, and 7% used a vessel of cold water. The majority (54%) walked to the market, 21% used public transportation, 18% drove a three-wheel drive, and 7% rode in an animal-drawn cart. For 36% of them, the time it took to get to the marketplace was less than 30 minutes, for 43% it took 30 minutes to 1 h and for 21% it took more than 1 h. Most respondents (97%) cleaned the cottage cheese containers regularly. The most frequent container washers (77%) used water and detergents, while the rest used different herbs. None of the factors outlined above were statistically significantly associated with contamination of cottage cheese with *Salmonella.*

#### Risk factors for cottage cheese contamination at the retailer level

The production system was the only variable in the value chain of cottage cheese retailers in this study that revealed statistical significance. Milk from peri-urban residents were 10 times more exposed to *Salmonella* than urban dwellers (85.6%; *p* = 0.009). Females comprised almost four-fifths of the respondents, while males were 20%. In informal, primary school, high school, preparatory, and diploma/degree, the educational background evaluation indicated 2.2%, 24.5%, 22.2%, 4.4%, and 46.7%, respectively. The respondents’ experience in years was less than a year, 1–2 years, 2–5 years, 5–10 years, and more than 10 years accounted for 17.8%, 18.9%, 42.2%, 8.9%, and 12.2% of the respondents, respectively.

The majority (77.8%) of individuals interviewed in this value chain did not train on how to handle and store cottage cheeses properly. A quarter of them (26.7%) walked to the market, 20% used public transportation, 15.6% drove three-wheel drives, 5.5% used four-wheels, 28.9% used refrigerated vehicles, and 6.7% traveled in animal-drawn carts. Over half of them (52.2%) kept cottage cheese in a separate refrigerator until sold. The majority (71.1%) had backup generators, which they used when the power went out.

## Discussion

Our findings demonstrated that though the prevalence of *Salmonella* contamination was highest in raw milk samples and milk samples collected from milk collectors, *Salmonella* contamination was detected in all tested sample types collected at all value chain points, and in all studied regions.

From a region perspective, the prevalence of *Salmonella* in samples collected from Oromia (14.8%) is consistent with previously published data by Beyene et al. (2016), Asfaw Ali et al. (2020), Abunna et al. (2017), and Tadesse and Dabassa (2012) potentially indicating that efforts on milk safety should be focused on milk production in this region. However, some reports demonstrated a much lower prevalence. For instance, the work of Dadi et al. (2020), Hailu et al. (2015), Liyuwork et al. (2013), Reta et al. (2016), Ejo et al. (2016), and Tadesse and Gebremedhin (2015), for *Salmonella* prevalence in Oromia region (Sebeta), Amhara region (Gondar), Addis Ababa, Somali region, and Amhara region (Gondar), respectively, were lower as compared to the current work result. Therefore, further surveillance may be necessary.

Our study indicated that the prevalence of *Salmonella* contamination was higher in raw milk, as compared to pasteurized milk. In contrast, the prevalence reported here was higher compared to past studies of Liyuwork et al. (2013) and Ejo et al. (2016), who recorded 2.1% and 0% *Salmonella* in pasteurized milk in Ethiopia, respectively. However, according to a report by CDC (1984), there was a 15% prevalence in this value chain, which was consistent with the current study. This high occurrence may be attributed to the samples’ insufficient pasteurization and post-pasteurization contamination (CDC, 2019). The higher prevalence of *Salmonella* in pasteurized milk suggests the possibility that some milk processors are carrying out pasteurization at temperatures that are lower than those recommended for effective pasteurization of milk or are pasteurizing milk for a shorter than recommended time. If pasteurization is effective, it will inactivate *Salmonella*; hence *Salmonella* will not be able to grow unless a post-pasteurization contamination has occurred and the milk storage conditions (e.g., temperature) supported its growth.

There are a limited number of reports on the prevalence of *Salmonella* in cottage cheese in Ethiopia. In 2013, Liyuwork and his colleagues reported 3.1% prevalence of *Salmonella* in tested cottage cheese samples collected in Ethiopia, which is consistent with our findings. The boiling process causes *Salmonella* to be more prevalent in raw milk than in cottage cheese. As a result, the bacteria cannot tolerate the heat and dies. Furthermore, the change in the pH and consistency of milk also alters the conducive environment that the bacteria used to live (Coelho et al., 2022).

Analysis of risk factors associated with *Salmonella* contamination of milk and cottage cheese has shown the critical points in which intervention may be most effective. An interesting finding was that a higher prevalence found among the milk producers, those who received training related to milk handling than those who did not. However, we do not know much about those trainings and there may be a need to evaluate the effectiveness of trainings in the future, suggesting more efforts to be made. However, without a fuller understanding of the curriculum in these trainings, this is simply a speculation. Another point could simply be that trainees are not applying—or cannot apply (due to limited resources)—the practices that were recommended in trainings. Therefore, future trainings should consider this data and implement methods to ensure adoption of training information, which could include pre- and post-assessments, follow up trainings, assessments of competencies, etc.

It is widely considered that hot water is preferred over cold water for sanitation, as it not only cleans but also kills bacteria. Oddly, these were not the findings of this study. In hot water, chemical processes that are important for cleaning happen more quickly because the heat helps destroy bacteria. The molecules of water move more quickly and bounce off one another more when it is heated. As a result, there is more room between the molecules for dissolved solvents to fill. Hot water may therefore dissolve a lot more substance than cold water (Al-Hubaety et al., 2013).

Additionally, this study found that containers in which milk is stored, or transported, should be kept clean: otherwise, they may impact the safety of the milk. In this study we found that aluminum can resulted in lower *Salmonella* contamination. This could be due to the ease of sanitation of such containers; however, this was not evaluated in this study.

Additionally, this data shows that refrigeration practices should be increased significantly, as it can help reduce microbial growth and allow for milk to be kept for a longer time. This information can guide specific point at which policymakers must intervene to ensure the safety of milk and its products. Otherwise, the severity of *Salmonella* contamination may continue to endanger humans and livestock and cause significant economic damage.

In this study, the practice of filtering milk by collectors upon reception resulted in an inverse association with *Salmonella* contamination. Previous studies have indicated that filtering can increase milk quality and microfiltration can remove microorganisms (Elwell and Barbano, 2006; Garcia et al., 2013), however, filtering can also introduce contaminants present on the material used for filtration. Furthermore, the pore size of the filter may allow microorganisms to pass through.

In this study, factors such as training on milk quality and safety, the use of hot or cold water to clean the udder of the animals, the practice of mixing the morning and night milk, the storage of milk containers, and the refrigeration of milk were significantly associated with *Salmonella* contamination. The conclusion from this point is that dairy farmers should not only focus on the money they earn for the production of milk but also protect the health of the community and themselves. Otherwise, salmonellosis will indeed take root and affect the health and economy of the community, reducing production and productivity.

## Conclusions

Generally, the results from this study indicate the safety of milk and other dairy product based upon the prevalence of *Salmonella* in all studied regions was poor. Milk composition, sanitation, and sanitary conditions of personnel working with milk and its products, with high regard for human health, might be the factors to contribute to the contamination of milk with *Salmonella*.

In conclusion, to improve the quality and safety of raw milk and milk products in Ethiopia, effective training and raising awareness among stakeholders at each level of the supply chain (producers, collectors, processing factories, and retailers/supermarkets) are necessary. Furthermore, public education regarding the hygienic practices, safety, and risks of consumption of raw or dairy products are important lines of defense against *Salmonella* infection and other food-borne pathogens transmitted through dairy products in the region.

## Data Availability

Not applicable.
